# Inhibitory Effects of Vinpocetine on the Progression of Atherosclerosis Are Mediated by Akt/NF-κB Dependent Mechanisms in apoE^-/-^ Mice

**DOI:** 10.1371/journal.pone.0082509

**Published:** 2013-12-09

**Authors:** Jianhui Zhuang, Wenhui Peng, Hailing Li, Yuyan Lu, Ke Wang, Fan Fan, Shuang Li, Yawei Xu

**Affiliations:** 1 Department of Cardiology, Shanghai Tenth People’s Hospital, Tongji University School of Medicine, Shanghai, China; 2 Department of Ophthalmology, Eye & ENT Hospital, Fudan University School of Medicine, Shanghai, China; Medical University Innsbruck, Austria

## Abstract

**Background:**

Recent studies have found additional roles for vinpocetine, a potent phosphodiesterase type I inhibitor, in anti-proliferation and anti-inflammation of vascular smooth muscle cells and cancer cells via different mechanisms. In this study, we attempted to investigate whether vinpocetine protected against atherosclerotic development in apoE^-/-^ mice and explore the underlying anti-atherogenic mechanisms in macrophages.

**Methodology/Principal Findings:**

Vinpocetine markedly decreased atherosclerotic lesion size in apoE^-/-^ mice measured by oil red O. Masson’s trichrome staining and immunohistochemical analyses revealed that vinpocetine significantly increased the thickness of fibrous cap, reduced the size of lipid-rich necrotic core and attenuated inflammation. *In* *vitro* experiments exhibited a significant decrease in monocyte adhesion treated with vinpocetine. Further, active TNF-α, IL-6, monocyte chemoattractant protein-1and matrix metalloproteinase-9 expression induced by ox-LDL were attenuated by vinpocetine in a dose-dependent manner. Similarly, ox-LDL-induced reactive oxygen species were significantly repressed by vinpocetine. Both western blot and luciferase activity assay showed that vinpocetine inhibited the enhanced Akt, IKKα/β, IκBα phosphorylation and NF-κB activity induced by ox-LDL, and the inhibition of NF-κB activity was partly caused by Akt dephosphorylation. However, knockdown of PDE1B did not affect Akt, IKKα/β and IκBα phosphorylation.

**Conclusions:**

These results suggest that vinpocetine exerts anti-atherogenic effects through inhibition of monocyte adhesion, oxidative stress and inflammatory response, which are mediated by Akt/NF-κB dependent pathway but independent of PDE1 blockade in macrophages.

## Introduction

Atherosclerosis is a chronic inflammatory process affecting the large- and medium-size arteries [1]. According to two typical structural determinants of vulnerability of atherosclerotic plaque, namely lipid-rich necrotic core and fibrous cap, atherosclerotic plaques are generally categorized as stable and unstable plaques, as unstable plaques possess larger of lipid-rich necrotic core and thinner of fibrous cap. It is also accepted that plaque instability, rather than plaque size, primarily accounts for the risk of thrombosis [2,3]. Plaque rupture and subsequent thrombotic occlusion of culprit vessels are the main cause of acute coronary syndrome or stroke. 

 Experimental studies have strongly supported that transmigration of monocytes into the subendothelial space and subsequent transformation into macrophage-derived foam cells are key events in atherogenesis [4–6]. Macrophages within atherosclerotic plaques secrete inflammatory cytokines, chemoattractants and matrix metalloproteinases (MMPs) as well, which not only promote atherosclerotic development but also lead to plaque rupture through degradating extracellular matrix and thinning of fibrous cap [7]. Furthermore, previous mechanistic studies consistently demonstrate that activation of classical signaling pathways, including PI3K/Akt, NF-κB and MAPK, driven by uptake of oxidative low density lipoprotein (ox-LDL) into macrophages during atheroprogression are responsible for the overexpression of inflammatory cytokines, chemoattractants and MMPs.

 Although the new drugs inhibiting inflammation or specially targeting MMPs offer an increasing valuable therapeutic strategy for preventing atherosclerotic progression and stabilizing rupture-prone plaques, their potential for clinical translation is limited due to either dubious effectiveness or intolerable adverse effects [8–10]. Phosphodiesterases (PDEs), categorized into 11 different families based on structural similarity, are intimately involved in the cardiovascular remodeling and atherogenesis by modulating cAMP and/or cGMP pathways [11–14]. Although the roles of PDE3 inhibitor (cilostazol) and PDE5 inhibitor (sildenafil) in the treatment of cardiovascular disorders have been extensively studied in clinical research [15,16], the roles of pharmacological agents that target other PDE members have not been investigated in detail. Vinpocetine is a semisynthetic derivative of vincamine, an alkaloid extracted from the periwinkle plant during the late 1960s [17,18]. It has been proved that vinpocetine is a PDE1 inhibitor which increases intracellular cGMP and cAMP contents [19], whereby activating protein kinase G (PKG) and protein kinase A (PKA) respectively. Voltage-dependent Na^+^ channels and Ca^2+^ Channels are also inhibited by vinpocetine [20,21]. Besides, vinpocetine relaxes cerebral smooth muscle cells and hence enhances cerebral blood flow [22]. Given these favorable effects in vasodilation and neuro-protection, vinpocetine has been widely used in the treatment of cerebrovascular disorders for decades [23]. More recently, vinpocetine was reported to prevent vascular smooth muscle cells (VSMCs) and breast cancer cells proliferation [24,25]. It was also suggested that vinpocetine inhibited inflammation in VSMCs via an IKK-dependent but PDE1-independent mechanism [26].

 Based on the above knowledge, we explored the effects of vinpocetine on plaque progression and morphology in apoE^-/-^ mice. *In vitro* experiments were also performed to investigate the roles of vinpocetine in these cellular and molecular processes.

## Materials and Methods

### 1: Ethnic statement

The experiments outlined in this manuscript conform to the Guide for the Care and Use of Laboratory Animals published by the National Institutes of Health (NIH Publication, 8th Edition, 2011). All animal procedures were approved by the Animal Care and Use Committees of Shanghai Tenth People’s Hospital.

### 2: Materials

Phorbol 12-myristate 13-acetate (PMA), low density lipoprotein (LDL), Oil Red O, paraformaldehyde, 3,3-diaminobenzidine tetrahydrochloride (DAB), vinpocetine and PI3K/AKT inhibitor LY294002 were purchased from Sigma (St. Louis, MO, USA). 2’, 7’-dichlorofluorescein diacetate (DCFH-DA), Trizol reagent, Hoechst 33342, CFSE and Lipofectamine 2000 were purchased from Invitrogen (Carlsbad, CA, USA). PrimeScript RT reagent Kit and SYBR Premix Ex Taq were purchased from Takara Biotechnology (Tokyo, Japan). Annexin V-FITC/PI Apoptosis Detection kit was purchased from eBioscience (San Diego, CA, USA). Lactate dehydrogenase (LDH) leakage assay was purchased from Beyotime (Shanghai, China). Direct cGMP ELISA kit was purchased from Enzo (Farmingdale, NY, USA). Dual-Luciferase Reporter Assay System and pGL4.32 [*luc2P*/NF-κB-RE/Hygro] vector were purchased from Promega (Madison, Wisconsin, USA). All the used primary antibodies (p-ERK1/2, t-ERK1/2, p-JNK1/2, t-JNK1/2, p-p38, t-p38, t-Akt, t-IKKα and t-IKKβ diluted 1:1000; p-IKKα/β, p-Akt and MCP-1 diluted 1:600; p-IκB diluted 1:300) were purchased from Cell Signaling Technology (Danvers, MA, USA), except for MMP-2 (diluted 1:500), TNF-α (diluted 1:300) and IL-6 (diluted 1:600) obtained from Santa Cruz (Santa Cruz, CA, USA) and MMP-9 (diluted 1:800) from Abcam (Cambridge, MA, USA).

### 3: *In vivo* experiments

#### Animals

Six-week-old male apoE^-/-^ mice on the C57BL/6 background strain (n = 22) were obtained from Vital River Laboratory (Beijing, China) and fed a high cholesterol diet containing 16.6% fat, 10.6% sucrose and 1.3% cholesterol (SLAC Laboratory Co. Ltd., Shanghai, China). Mice were intraperitoneally injected with 5mg/kg vinpocetine as previous described [24,26] or the same dosage of saline solution every day for 12 weeks from 6 weeks of age. Body weight and food-intake were monitored every week throughout the treatment period in both groups.

#### Plasma glucose and lipid panel analysis

Plasma samples (250 μl) from C57BL/6 and apoE^-/-^ mice, fasted for 12 h before euthanasia, treated with either vehicle or vinpocetine were collected. Fasting glucose, total cholesterol and triglyceride, low-density lipoprotein cholesterol (LDL-C) and high-density lipoprotein cholesterol (HDL-C) were measured by colorimetric enzymatic assay systems (Roche MODULAR P-800, Swiss Confederation).

#### Histological analysis

After 12 weeks of treatment, animals were fasted for 12 h prior to euthanasia, then anesthetized by intraperitoneal injection of 3% pentobarbital at a concentration of 70 mg/kg and scarified. The heart and aorta were removed after *in situ* perfusion with PBS followed by 4% paraformaldehyde. After fixation for 1 day in 4% paraformaldehyde, 4 μm-thick paraffin-embedded cross-sections of aortic root were prepared for the following histology and immunohistochemical analyses.

 Lesion development was measured using *en face* method that aortic arch and thoracic aorta were stained with Oil Red O as previously described. The cross-sections were stained with hematoxylin and eosin to evaluate the atherosclerotic lesion complexity or with Masson’s trichrome to evaluate the presence of collagen. Collagen was quantified by calculation of the proportion of area occupied by the Masson’s trichrome staining within the plaques by Image-Pro Plus 6.0 (Media Cybernetics Inc., MD, USA).

#### Immunohistochemical analysis

For immunohistochemical staining, cross-sections were labeled with a rabbit polyclonal antibody against MMP-9 (diluted 1:400) and mouse monoclonal antibody against TNF-α (diluted 1:100) at 4°C overnight after microwave antigen retrieval in citrate buffer. After washing, the bound antibodies were conjugated with species-specific secondary antibodies at 37°C for 1 hour and then incubated with DAB substrate for 1-3 minutes. Specimens were counterstained with hematoxylin and images were acquired with Leica DMI6000 microscopy (Leica, Germany). Three sections from each animal were stained for mean values, which were expressed as a percentage of total lesion area. Data represent means ± S.E.M. from six animals in each group.

### 4: *In vitro* experiments

#### Preparation of ox-LDL, cell culture and transfection

Preparation of ox-LDL was performed as previously described [27]. After dialysis against PBS for 24 h at 4°C, oxidation of LDL was performed in the presence of 5 μM CuSO_4_ in PBS for 24 h in a water bath at 37°C, followed by extensive dialysis to remove Cu^2+^. The extent of LDL oxidation was determined by measuring thiobarbituric acid-reactive substances (TBARs). Ox-LDL containing 30–60 nmol of TBARs defined as malondialdehyde equivalents per milligram of LDL protein was used.

 Human monocytic THP-1 cells were purchased from Institute of Biochemistry and Cell Biology in Shanghai and cultured as previously described [28]. To differentiate into macrophages, THP-1 cells were cultured in the presence of 100 nM PMA for 48 hours. After pretreatment of vinpocetine for 1 hour, PMA-induced macrophages were then incubated with ox-LDL (20 μg/ml) for 12 hours and harvested for the following experiments.

 The duplex small interfering RNA (siRNA), targeting PDE1B mRNA (forward sequence: GGAUCUUCGUGGAACGGAUTT; and reverse sequence: AUCCGUUCCACGAAGAUCCTT), and a negative control (scramble sequence) were purchased from GenePharma (Shanghai GenePharma Co. Ltd., China). PMA-treated THP-1 cells were transfected with 100nM siRNA using Lipofectamine 2000 for 24 h. The expression of PDE1B after transfection was confirmed by RT-PCR.

#### Analysis of monocyte-endothelial cell adhesion

Human umbilical vein endothelial cells (HUVECs), purchased from Clonetics Cell Discovery Systems (San Diego, CA, USA) were grown in DMEM (Gibco, NY, USA) containing 10% fetal bovine serum in a humidified incubator (37°C, 5% CO_2_) and used between passage 3 and 10. For the monocyte-endothelial cell adhesion analysis, HUVECs was grown to confluence in 6-well plates and treated with 20 μg/ml ox-LDL in the presence or absence of vinpocetine for 24 h. After respective incubation of HUVECs stained with Hoechst 33342 and THP-1 monocytes with CFSE for 20 min, THP-1 monocytes labeled with CFSE was added to each well in the same conditions for 1 h. The number of adherent cells was expressed as percentage of positive cells double stained with CFSE and Hoechst 33342.

#### Cell viability and apoptosis

The measurement of LDH activity in the extracellular medium was used to determine cell viability according to the manufacture’s protocol. The loss of intracellular LDH and its release into the culture medium is an indicator of irreversible cell death due to cell membrane damage. After treatment of cultured macrophages and HUVECs with different concentrations of vinpocetine for 12 h, 100 µL cell medium was collected for LDH activity analysis and the absorbance at 490 nm was measured by a automatic microplate reader.

Analysis of apoptotic HUVECs was determined after Annexin V-FITC-PI staining by flow cytometry (FACSCalibur, BD Biosciences, San Jose, USA). Cells displaying only Annexin V positive staining were considered as early apoptosis, while cells stained with Annexin V and PI were late apoptosis.

#### Detection of foam cell formation

Macrophages were incubated with 20 μg/ml ox-LDL in the presence or absence of vinpocetine for 24 h. After fixation of 4% paraformaldehyde for 15 minutes, cells were washed with 60% isopropyl alcohol and stained with 0.2% Oil Red O solution. Cells were examined by light microscopy (×400).

#### Measurement of intracellular ROS

ROS production, including H_2_O_2_ and other peroxides, in macrophages was quantified using the fluorescent probe DCFH-DA. After treatment, PMA-treated THP-1 cells (1×10^6^) were labeled with 0.5 nM DCFH-DA for 30 min at 37°C. The fluorescence intensity of ROS was measured by flow cytometry.

#### Gelatin zymography

MMP-2 and MMP-9 activity was measured by gelatin zymography. Briefly, 12 μl aliquots culture supernatants were collected and loaded onto 10% polyacrylamide gel containing 1 mg/ml gelatin and electrophoresed. After electrophoresis, gels were rinsed twice in 2.5 % Triton X-100 to remove SDS. Gels were then incubated in substrate buffer (50 mM Tris-HCl, 5 mM CaCl_2_, 1 μM ZnCl_2_ and 0.02% Brij-35), followed by staining with 0.05% Coomassie Blue R-250 for 3 hours and scanning with Odyssey imaging system (Li-cor, NE, USA).

#### Dual-Luciferase reporter assay

Cells seeded in 12-well plates (8×10^5^) were transiently transfected with pGL4.32 [*luc2P*/NF-κB-RE/Hygro] vector using Lipofectamine 2000. Transfected cells were serum-starved for 36 hours, followed by pretreatment of vinpocetine for 1 hour and exposure to ox-LDL for 12 hours. NF-κB luciferase activity was measured using Dual-Luciferase Reporter Assay and normalized to *Renilla* luciferase activity according to the manufacture’s introduction.

#### Western blot analysis

Protein extraction and western blot analysis was performed as previously described in our study [28]. In briefly, 20 μg proteins were separated by SDS-polyacrylamide gel electrophoresis and transferred to nitrocellulose membranes. After blocking, the filters were incubated with the following antibodies at 4°C overnight: p-ERK1/2, t-ERK1/2, p-JNK1/2, t-JNK1/2, p-p38, t-p38, t-Akt, t-IKKα and t-IKKβ (diluted 1:1000), p-IKKα/β, p-Akt and MCP-1 (diluted 1:600), p-IκB diluted 1:300 (Cell Signaling Technology, Danvers, MA, USA); MMP-2 (diluted 1:500), TNF-α (diluted 1:300) and IL-6 (diluted 1:600) (Santa Cruz, CA, USA); and MMP-9 (diluted 1:800) (Abcam, Cambridge, MA, USA). After washing and incubation with anti-rabbit or anti-mouse secondary antibodies IgG (Amersham) for 1 hour, membranes were visualized with Odyssey imaging system (Li-cor, NE, USA).

#### RNA isolation and quantitative reverse-trasnscript polymerase chain reaction (RT-PCR)

Total RNA was extracted from THP-1 macrophages using Trizol reagent and from peripheral mononuclear blood cells (PBMCs) of five healthy participants using an RNeasy Mini Kit (Qiagen, Hilden, Germany) as previously described [29]. Reverse transcription was performed using 1 μg of RNA with a PrimeScript RT reagent Kit and the relative mRNA expression levels were determined by RT-PCR using SYBR Premix Ex Taq assay. Primer sequences used in RT-PCR are documented in Table S1.

#### Measurements of intracellular cGMP

Quantitative determination of cGMP in PMA-treated THP-1 was performed according to the manufacture’s introduction. Briefly, cell lysates in 12-well plates were harvested with 300 μl HCl at a concentration of 0.1M. After collection, the samples mixed with 50μl Neutralizing Reagent were pipeted into the bottom of appropriate wells and incubated with cGMP primary antibody and antigen conjugate at room temperature for 2 h. The plates were washed three times, leaving only bound cGMP. Then pNpp substrate solution and stop solution were added. The intracellular cGMP content was assessed by measuring absorbance at 405 nm using ELISA reader and calculating from a standard curve.

### 5: Statistical analysis

All of the analyses were performed with SPSS 14.0 (SPSS Inc., Chicago, IL, USA). Data were given as mean ± SEM unless otherwise indicated. Differences between two groups were examined using Student *t* test. One-way ANOVA was used to compare multiple groups, if appropriate, with Bonferroni correction for post hoc analysis. A *P*-value < 0.05 was considered statistically significant and < 0.005 was taken as significance for post-hoc analysis. All experiments were performed at least three times.

## Results

### Vinpocetine suppresses atherosclerotic development in apoE^-/-^ mice

**Figure 1:**
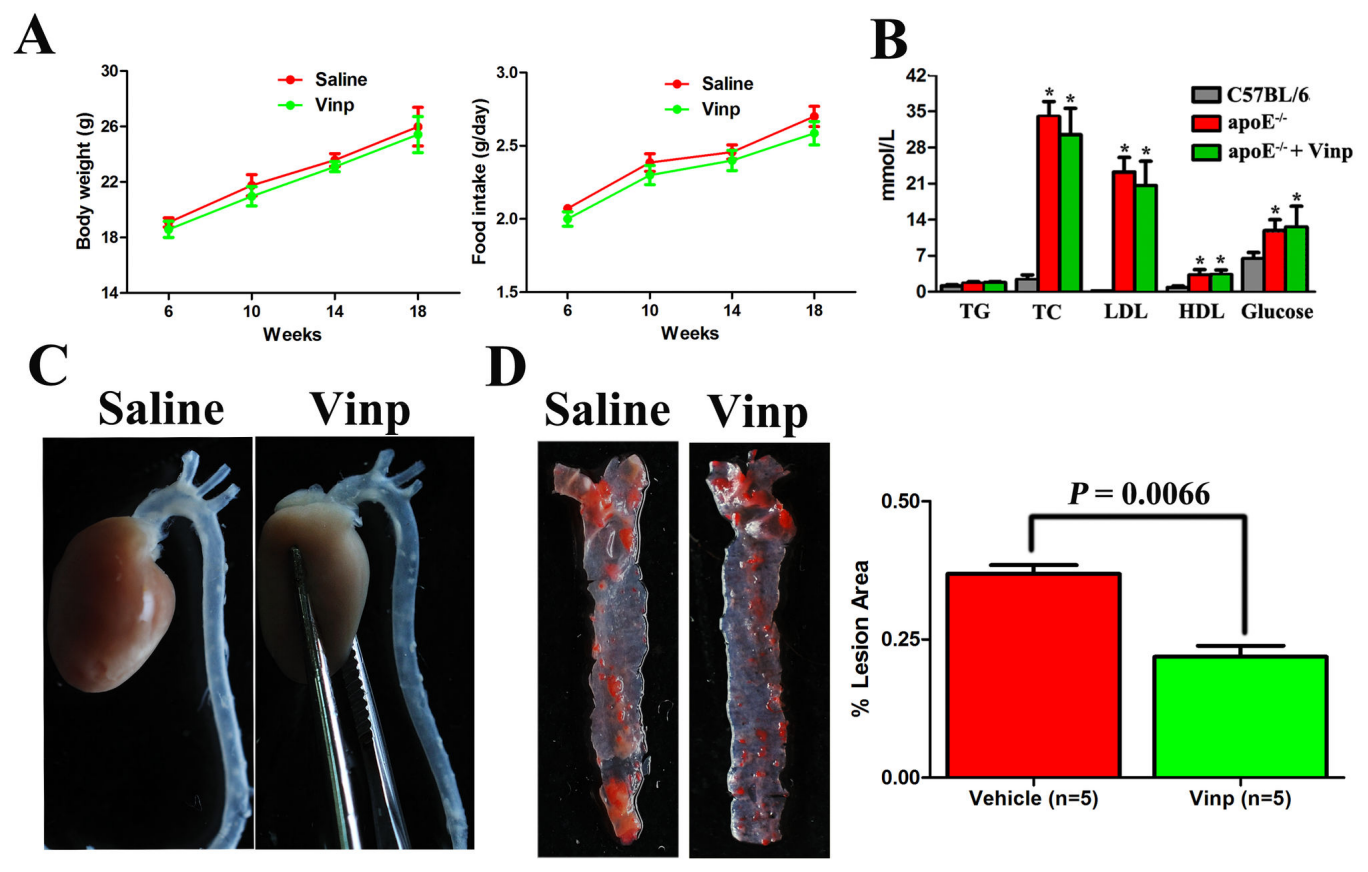
Vinpocetine suppresses atherosclerotic development in apoE^-/-^ mice. A. Comparisons of body weight and food intake in apoE^-/-^ mice treated with saline and vinpocetine (5mg/kg/d). B. Plasma levels of total cholesterol (TC), total triglyceride (TG), low-density lipoprotein cholesterol (LDL-C), high-density lipoprotein cholesterol (HDL-C) and glucose after 12 weeks of fat feeding. * *P* < 0.05 vs. C57BL/6 group. C. Representative photographs of gross examinations of plaque burden located at aortic arch and thoracic aorta. D. *En* *face* plaque quantification of aortic arch and thoracic aorta stained with oil red O. Data are obtained from five mice from each group and bars indicate mean ± SEM.

Vinpocetine treatment did not influence body weight and food intake in apoE^-/-^ mice during 12-week feeding (Figure 1A). Circulating levels of total cholesterol, LDL-C, HDL-C and glucose were remarkably increased in both groups of apoE^-/-^ mice relative to those in normal-fed C57BL/6 mice, but without differences between saline- and vinpocetine-treated groups (Figure 1B).

 Gross examination of the aorta revealed that vinpocetine-treated apoE^-/-^ mice had decreased atherosclerotic lesion area compared with saline-treated apoE^-/-^ mice (Figure 1C). The aortic arch and the thoracic aorta were longitudinally cut open and stained *en face* with oil red O to detect lipid deposition, after which the lesions were measured as percentage lesion area. As delineated in Figure 1D, vinpocetine treatment significantly decreased longitudinal lesion development in apoE^-/-^ mice compared with saline-treated apoE^-/-^ mice (lesion area 19.63 ± 2.73% vs. 31.78 ± 1.92%; *P*=0.0066, n = 5).

### Vinpocetine stabilizes atherosclerotic plaque and attenuates inflammation in apoE-/- mice

**Figure 2:**
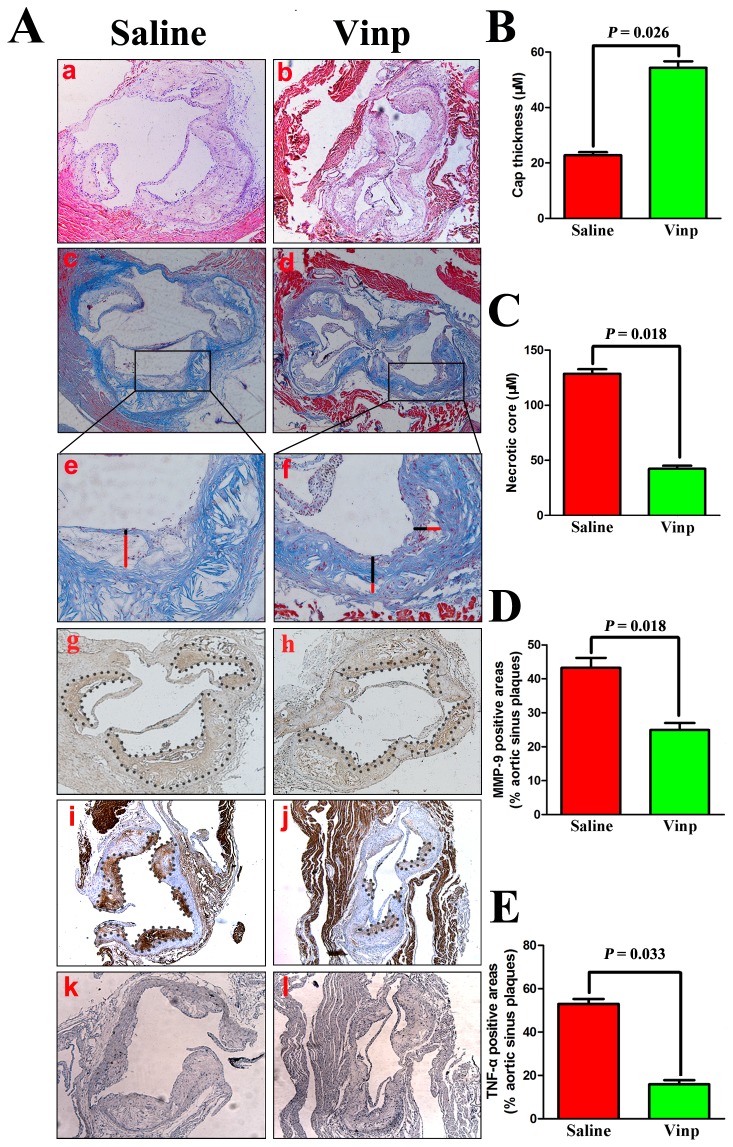
Vinpocetine affects plaque composition in apoE^-/-^ mice. A. Representive pictures of hematoxylin and eosin staining (a-b), Masson’s trichrome staining (c-f), MMP-9 (g-h), TNF-α (i-j) and normal IgG (k-l) of atherosclerotic plaques in aortic sinus. Through immunohistochemistry, brown staining exhibits positive area, while blue represents counterstaining with hematoxylin. Absolute values of necrotic core size (B) and cap thickness (C) with max depth in local plaques from the aortic sinus and representative images (e and f) to show how the necrotic core (red line) and fibrous cap (black line) in plaques from the aortic sinus was measured. Results represent the percentage of areas occupied by MMP-9 (D) and TNF-α (E) versus total plaque area within aortic sinus. Immunohistochemical staining of normal IgG (k-l) was presented as negative control experiments. Data are obtained from six mice from each group and bars indicate mean ± SEM.

With regard to plaque stabilization, Masson trichrome staining revealed that vinpocetine markedly enhanced collagen content and fibrous cap thickness in the aortic sinus. Conversely, lipid-rich necrotic core size was significantly decreased after vinpocetine treatment (Figure 2A-C). Considering other aspects of plaque composition, immunohistochemistry analysis showed that reduced expression of TNF-α and MMP-9 were detected in the aortic sinus of vinpocetine-treated apoE^-/-^ mice (*P*=0.033 and *P*=0.018, respectively) (Figure 2A g-j, 2D and 2E).

### Vinpocetine suppresses monocytes recruitment but does not affect foam cell formation

**Figure 3:**
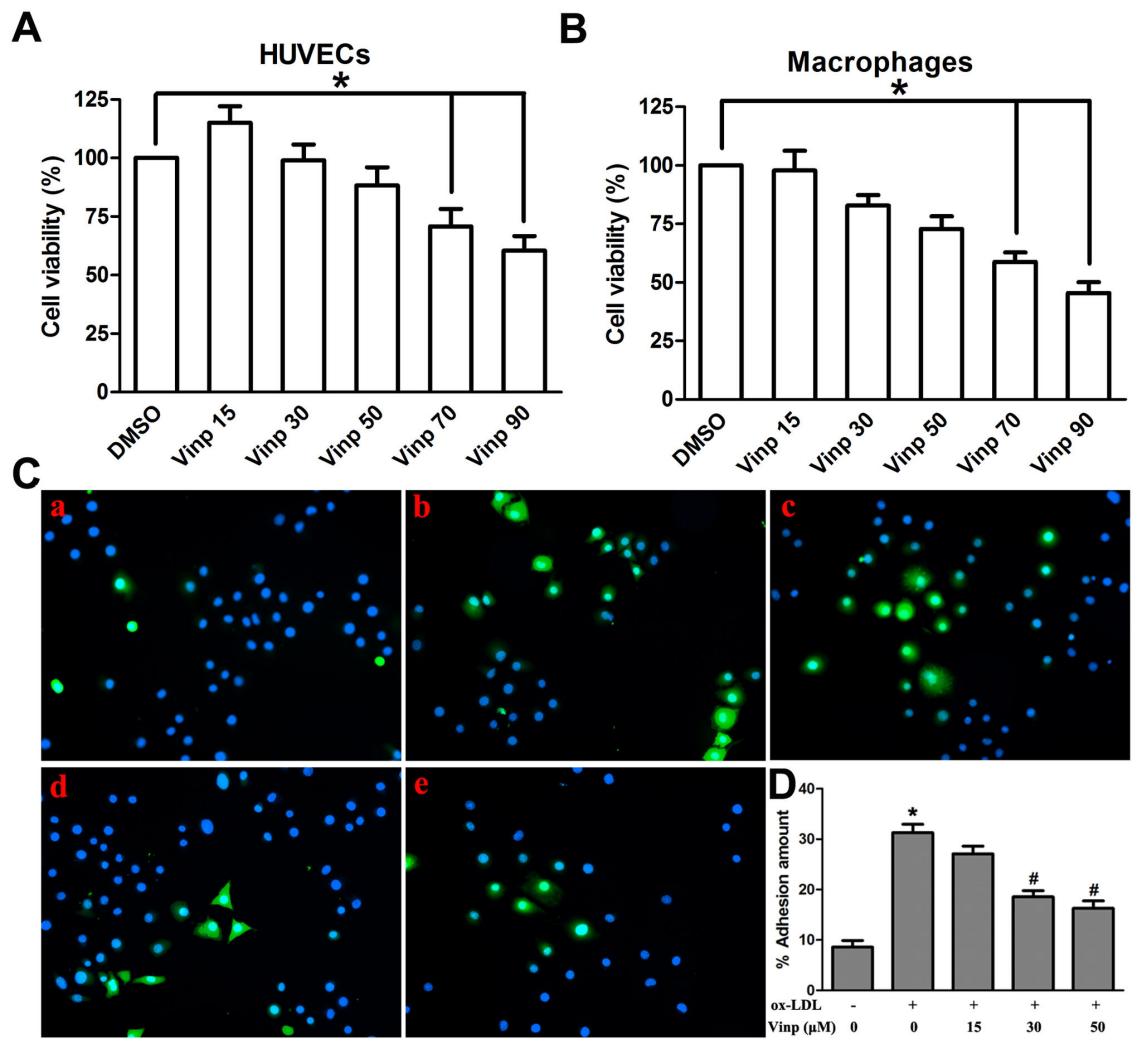
Effects of vinpocetine on cell viability and monocyte adhesion. A and B. Effects of vinpocetine on cell viability were assessed using the LDH leakage assay. C. Representative photos of CFSE-labeled adherent cells were measured by fluorescence microscope. Hoechst 33342 was used as a nuclear stain of HUVECs. D. The number of adherent cells was expressed as percentage of positive cells double stained with CFSE and Hoechst 33342. (a. Control, b. ox-LDL, c. ox-LDL+vinp 15 μM, d. ox-LDL+vinp 30 μM, e. ox-LDL+vinp 50 μM). Note that the concentration of ox-LDL was 20 μg/ml. Experiments were performed in triplicate. Bars indicate mean ± SEM. * *P* < 0.005 vs. control. # *P* < 0.005 vs. ox-LDL alone.

First, we performed LDH leakage assay and Annexin V-FITC/PI apopotosis double staining to explore an appropriate range of concentrations and exclude the influence of apoptotic and toxic effects caused by vinpocetine. Figure 3A and B show vinpocetine significantly reduced HUVEC and macrophage viability at concentrations of 70 and 90 μM. In addition, vinpocetine did not aggregate or mitigate ox-LDL-induced HUVECs apoptosis in a range of concentrations from 15 to 50 μM (Figure S1). According to the above two results, we used 15, 30 and 50 μM as appropriate concentrations in the subsequent experiments.

To explore whether the inhibitory effect of vinpocetine on macrophage accumulation was attributed to a reduction of monocyte adhesion to ox-LDL stimulated endothelium, we performed the monocyte-endothelial cell adhesion assay. As shown in Figure 3C and D, vinpocetine blocked excessive monocyte adhesion induced by ox-LDL in a dose-dependent manner. To examine whether vinpocetine inhibited foam cell formation and lipid accumulation in macrophages, THP-1 macrophages were preincubated with ox-LDL in the presence or absence of vinpocetine for 12 hours, followed with Oil Red O staining. Nevertheless, it was found that vinpocetine did not effectively decrease lipid accumulation in cytoplasmic droplet caused by ox-LDL incubation (Figure S2).

### Vinpocetine inhibits ox-LDL-induced oxidative stress and inflammation in macrophages

**Figure 4:**
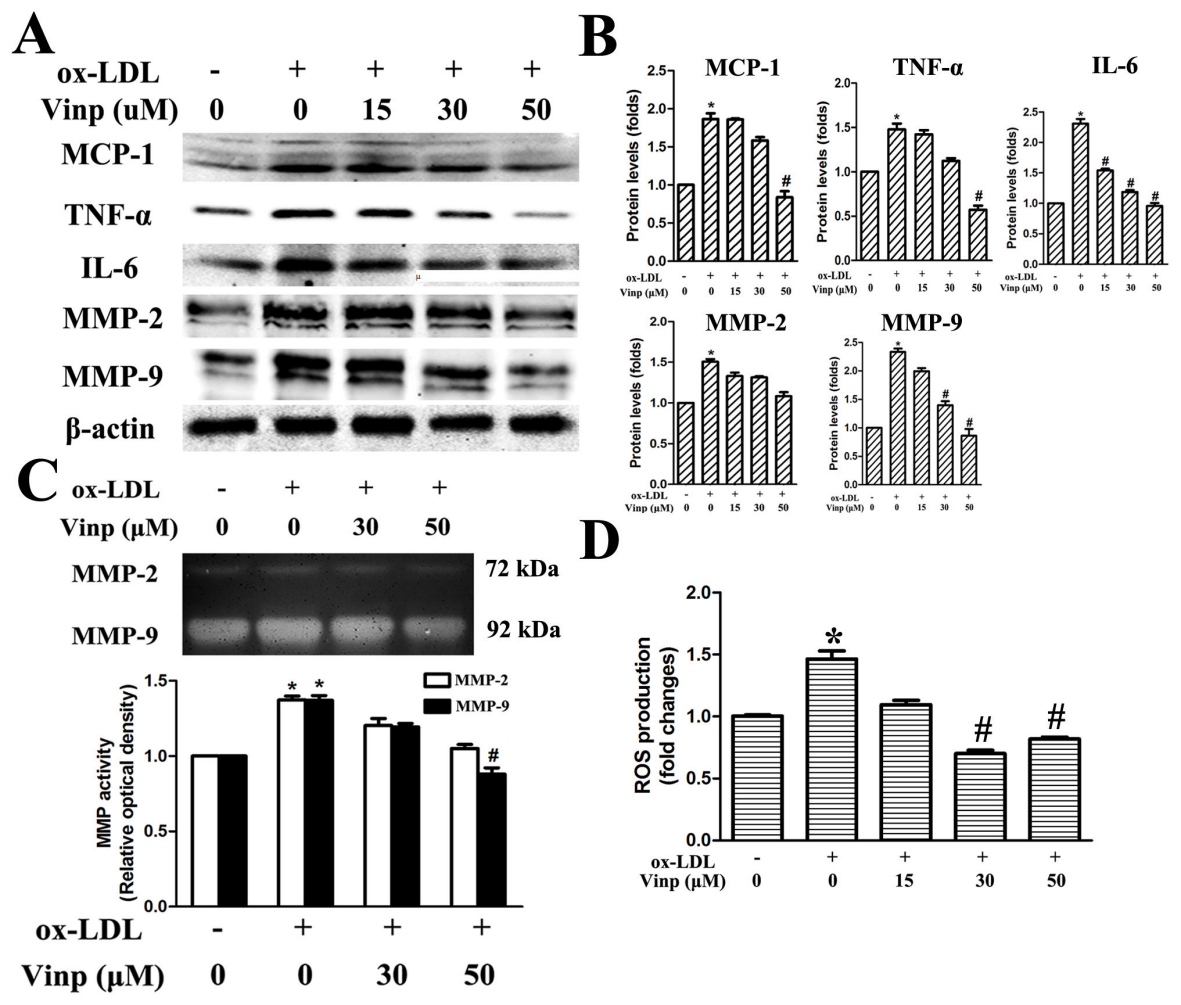
Inhibitory effects of vinpocetine on inflammation and oxidative stress in macrophages. A and B. Effects of vinpocetine on TNF-α, IL-6, MCP-1, MMP-2 and MMP-9 protein expression. C. MMP-2 and MMP-9 activity assessed by gelatin zymography. D. Relative levels of ROS productions after vinpocetine treatment. Human monocytic THP-1 cells were incubated with PMA (100 nM) for 48 h in order to differentiate into macrophages, followed with exposure to 20 μg/ml ox-LDL in the presence or absence of vinpocetine for 24 h. Experiments were performed in triplicate. Bars indicate mean ± SEM. * *P* < 0.005 vs. control. # *P* < 0.005 vs. ox-LDL alone.

It has been known that oxidative stress and inflammatory response in macrophages are intimately involved in lesion development and tightly associated with plaque rupture [30]. As shown in Figure 4, proinflammatory cytokines (TNF-α and IL-6) and chemoattractant MCP-1 were overexpressed in response to ox-LDL incubation, but were profoundly inhibited by pretreatment of vinpocetine in a dose-dependent manner. Western blot and gelatin zymography were performed to confirm the aforementioned observations that vinpocetine stabilized atherosclerotic plaques via downregulation of MMP-9 secreted by macrophages, showing that vinpocetine reduced ox-LDL-induced MMP-9 protein production and also enzymatic activity, while MMP-2 expression and activity retained comparable results but were moderately decreased by treated with vinpocetine at a concentration of 50 μM (Figure 4A-C). Likewise, ox-LDL (20 μg/ml) prominently induced the production of ROS in macrophages, and the production was partially suppressed by vinpocetine (Figure 4D).

### Vinpocetine inhibits ox-LDL-induced NF-κB activation through suppression of Akt, IKKα/β and IκB phosphorylation

**Figure 5:**
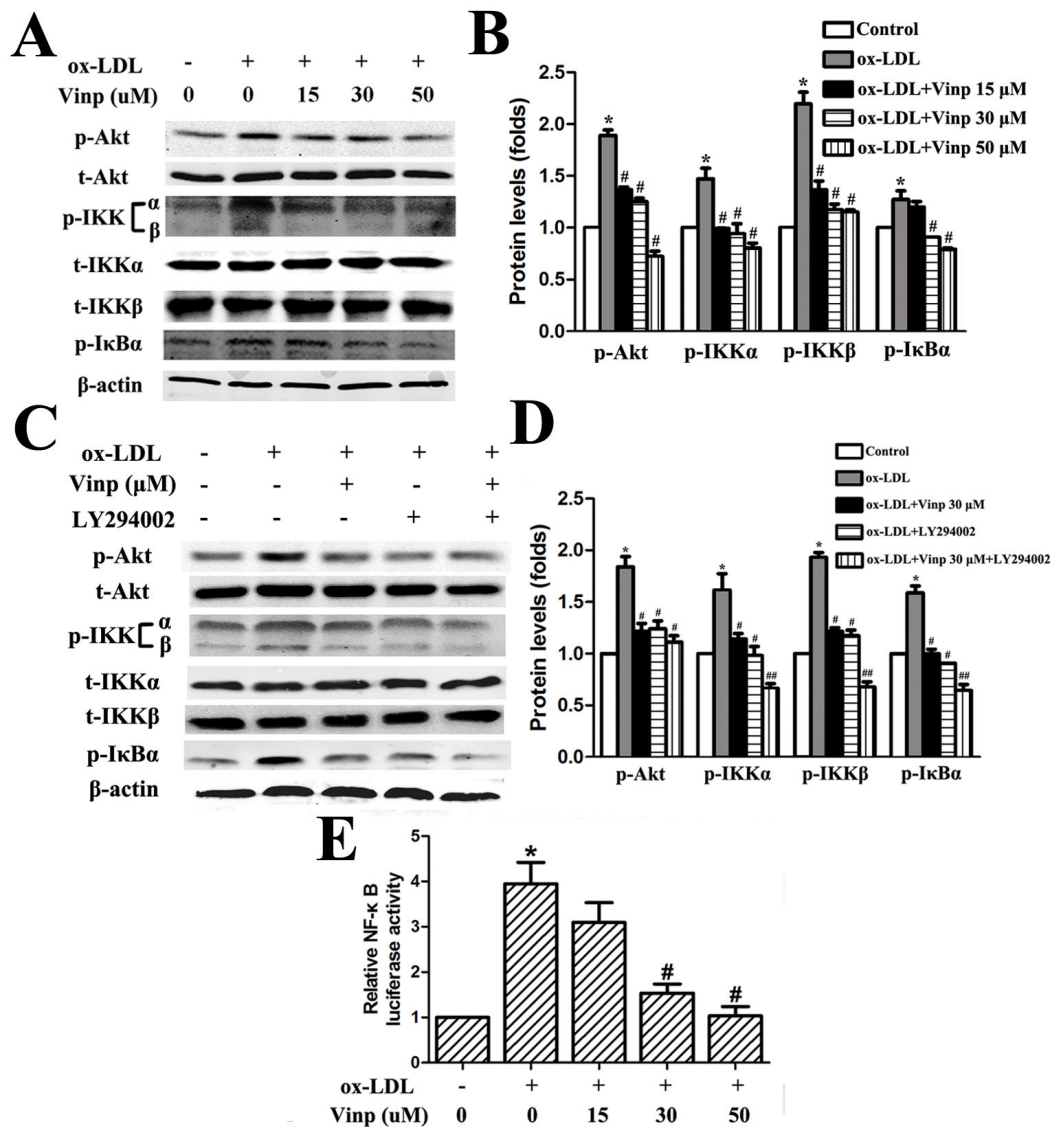
Vinpocetine inhibits ox-LDL-induced Akt, IKKα/β and IκB phosphorylation and NF-κB activity in macrophages. A and B. Effects of vinpocetine on ox-LDL-induced phosphorylation of Akt, IKKα/β and IκB. C and D. Effects of vinpocetine (30 μM) and LY294002 (10 μg/ml) treatment, alone or in combination, on ox-LDL-induced phosphorylation of Akt, IKKα/β and IκB. E. Dual-Luciferase reporter assay was performed to determine NF-κB activity. PMA-induced macrophages were transfected with pGL4.32 [*luc2P*/NF-κB-RE/Hygro] vector for 24 hours, then pretreated with vehicle and vinpocetine at indicated concentration for 1 h and followed with ox-LDL incubation for 12 hours. Each experiment was repeated four times. Bars indicate mean ± SEM. * *P* < 0.005 vs. control. # *P* < 0.005 vs. ox-LDL alone.

Inspired by previous studies [25,26] and our above observation, we sought to ascertain whether vinpocetine exerted anti-inflammatory effects through antagonism of Akt and NF-κB in macrophages. It is known that IKKα/β activation depending on the phosphorylation of the residues catalyzes IκB phosphorylation and degradation, which in turn releases NF-κB into nucleus. In the present study, stimulation of IKKα/β and IκB phosphorylation in response to ox-LDL incubation could be remarkably suppressed by vinpocetine treatment, ensuing inactivation of NF-κB (Figure 5A, B and E). Similarly, vinpocetine treatment concentration-dependently decreased the phosphorylation levels of Akt.

 We then explored the possibility that dephosphorylation of Akt by vinpocetine treatment was required for suppression of IKKα/β and IκB phosphorylation induced by ox-LDL in macrophages. As shown in Figure 5C and D, the inhibitory effects of vinpocetine at concentration of 30 μM on Akt, IKKα/β and IκBα phosphorylation could be mimicked by adding LY294002 (10 μg/ml), a specific Akt inhibitor. Furthermore, treatment with vinpocetine and LY294002, either in combination or alone, analogously repressed Akt phosphorylation by 1.5-fold, while vinpocetine plus LY294002 more strongly dephosphorylated both IKKα/β and IκBα than vinpocetine treatment alone. These results implied a synergistic contribution of vinpocetine and LY294002 to NF-κB inactivation. Taken together, the inhibitory effects of vinpocetine on NF-κB cascade were partly mediated by Akt dephosphorylation.

 Consistent with the results from previous studies [25,26], active phosphorylation of MAP kinases in response to ox-LDL stimulation, including ERK1/2, JNK1/2 and p38, was unaffected by vinpocetine treatment (Figure S3).

### Inhibitory effect of vinpocetine on NF-κB pathway is independent of PDE-1

**Figure 6:**
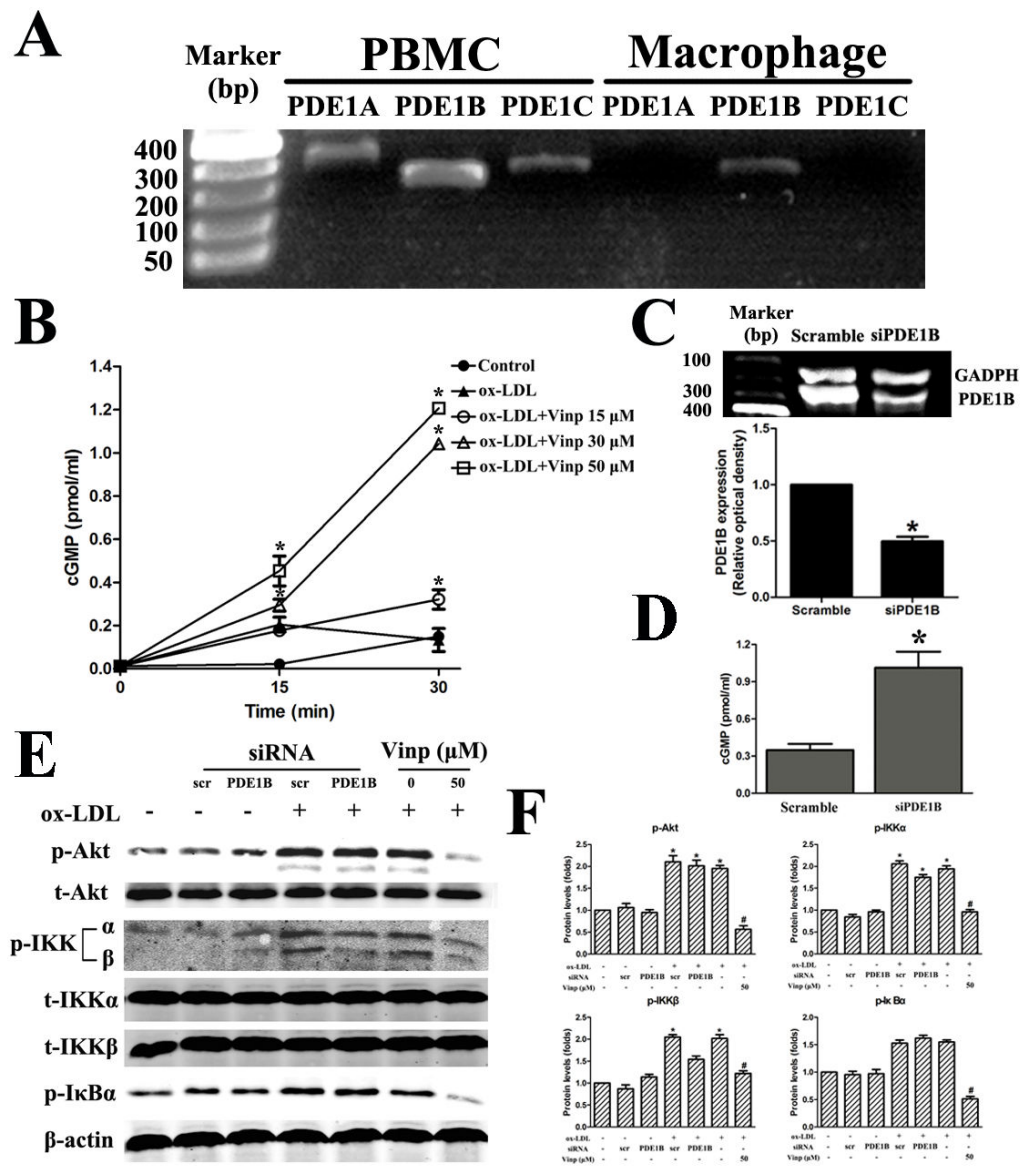
Inhibitory effects of vinpocetine on Akt/IKKα/β/IκB phosphorylation are independent of a PDE1B-mediated cGMP increase mechanism. A. Total RNA is isolated from peripheral blood mononuclear cells (PBMCs) or macrophages, and the expression of PDE1B is determined by PCR. B. Positive effect of vinpocetine on intracellular cGMP contents. PMA-induced THP-1 macrophages are pretreated with various doses of vinpocetine or vehicle for 15 and 30 min. C. The expression levels of PDE1B after transfection with scramble and PDE1B siRNA are determined by PCR. GAPDH indicates house-keeping gene glyceraldehyde-3-phosphate dehydrogenase. D. Knockdown of PDE1B leads to an increase in intracellular cGMP contents. E and F. Knockdown of PDE1B does not alter the phosphorylation levels of Akt, IKKα/β and IκB. Each experiment is repeated four times. Bars indicate mean ± SEM. 0 indicates vehicle DMSO. * *P* < 0.005 vs. control. # *P* < 0.005 vs. ox-LDL + vehicle.

Given that vinpocetine is a potent PDE1 inhibitor, it seems plausible that inactivation of NF-κB is mediated by PDE1-dependent mechanisms. Toward this end, we firstly examined whether three PDE1 isoforms, PDE1A, PDE1B and PDE1C, were expressed in THP-1 macrophages. Using RT-PCR, we found that PDE1B was the predominant isoform in THP-1 macrophages whereas the expression of PDE1A and PDE1C was undetectable compared with peripheral blood mononuclear cells (Figure 6A). When THP-1 macrophages were treated with vinpocetine, the intracellular cGMP levels were gradual increased in a dose-dependent manner at 15 and 30 minutes (Figure 6B). As expected, knockdown of PDE1B by siRNA resulted in an increase of intracellular cGMP contents (Figure 6C and D). Nevertheless, knockdown of PDE1B did not alter phosphorylation of Akt, IKKα, β and IκBα (Figure 6E and F).

## Discussion

Atherosclerosis is a chronic disease process with a recognized multifactorial etiology, ultimately developing rupture-prone plaques and leading to atherothrombotic events [1,3,31,32]. Clinical trials and animal experiments to date convincingly demonstrate that advance plaques share common properties: focal inflammation, thin fibrous cap, augmented lipid-rich necrotic core, macrophage accumulation and extracellular matrix degradation [3]. On the other hand, a prior study has identified vinpocetine as a promising anti-inflammatory candidate for the treatment of inflammatory diseases [26]. However, there is a clear lack of *in vivo* data supporting its inhibitory effects on atherosclerosis. In the present study, we demonstrate the marked effects of vinpocetine on preventing atherosclerotic development in apoE^-/-^ mice. Interestingly, serum glucose and lipid levels were not significantly altered by vinpocetine, suggesting that the inhibitory effects of vinpocetine on lesion growth were independent of glucose and lipoprotein metabolism and might be directed to plaque composition. Despite a minority event emerging during atheroprogression, the rupture or erosion of vulnerable plaques is fatal and clinical relevant to the great majority of symptomatic coronary thrombi [2]. Therefore, antagonists against plaque instability prove more valuable for preventing acute coronary thrombosis. In the present study, we found that the influence of vinpocetine on plaque morphology involved strengthening fibrous cap, reducing lipid-rich necrotic core size, decreasing TNF-α and MMP-9 expression based on the histological and immunohistochemistrical observations. These changes in plaque phenotypes comprehensively supported the benefits of vinpocetine to plaque stability. 

 Macrophages often accumulate in the shoulder region of vulnerable plaque and are certainly the main source of cytokines within plaques [6,33]. During the last decade, ox-LDL and its interaction with monocytes/macrophages have been considered as primary atherogenic components of dyslipidemia [34]. The concentration of ox-LDL is markedly elevated in atherosclerotic lesions and reaches cytotoxic levels that can stimulate monocyte recruitment, generate ROS and release proinflammatory cytokines as well [35]. Thus we exposed endothelial cells to ox-LDL in order to mimic the changes seen in atheroclerostic lesions, finding that vinpocetine acts on monocyte adhesion through inhibition of chemoattractant MCP-1 expression. Despite weak inhibitory potential towards foam cell formation, vinpocetine dramatically diminished ROS and proinflammatory cytokines expression in macrophages. Once monocyte adhesion initiates atherogenesis, subsequent foam cell formation paired with oxidative stress and inflammatory process last throughout plaque development and rupture. Interestingly, both immunoblotting and zymography showed that vinpocetine could inhibit MMP-9 expression and activity, yet not markedly influence MMP-2. The discrepancy can be explained by the fact that MMP-9, which predominates over MMP-2 in human macrophages, is preferentially overexpressed and activated in inflamed, vulnerable plaques compared to fibrous plaques [7,36]. Since MMP-9 functions as a promoter of matrix degradation, it is thus presumably that the protective effects of vinpocetine on plaque stabilization are partly due to the down-regulation of MMP-9.

 Previous studies accumulated over decades propose that activation of several well-known pathways, including PI3K/Akt, NF-κB and MAPK, and inflammatory response are largely the results of cumulative effect of ROS. Simultaneously, with regard to the mechanisms by which vinpocetine exerts anti-inflammatory actions, Jeon et al. [26] showed the anti-inflammatory effects of vinpocetine by directly targeting IKKβ. Other mechanistic studies referring to breast cancer cells and VSMCs demonstrated that vinpocetine could lead to cell cycle arrest because of an inhibition of Akt or ERK1/2 phosphorylation [24,25]. In line with these findings, we confirmed that vinpocetine could profoundly decrease the phosphorylation levels of Akt, IKKα/β and IκBα in macrophages. Additionally, the inhibition of IKKα/β phosphorylation offers reasonable supplementary information for the process that vinpocetine directly binds to IKK complex and then blocks its activity.

 Another intriguing observation finding that ox-LDL-stimulated IKKα/β and IκBα phosphorylation was inhibited by LY294002 recognized Akt as an upstream activator of NF-κB pathway. It should be noted that the involvement of PI3K/Akt signaling pathway in NF-κB activation is a cell-specific event [37,38]. In ox-LDL incubated macrophages, the active Akt phosphorylates the IKKα/β complex, which in turn phosphorylates IκBα for degradation. Moreover, vinpocetine and LY294002 worked synergistically in the dephosphorylation of IKKα/β and IκBα, suggesting that vinpocetine suppress NF-κB activity, at least in part, through suppression of Akt phosphorylation.

 Vinpocetine is well known to enhance cerebral circulation and cognitive function via PDE1-dependent mechanisms [39]. There are three subfamilies of PDE1, alias PDE1A, PDE1B and PDE1C. While PDE1C hydrolyze cAMP and cGMP with equal efficiency, PDE1A and PDE1B show high affinity for cGMP. Emerging evidence propose that both PDE1-denpendent and -independent mechanisms are involved in the regulation of VSMCs proliferation [11,13,24]. To our best knowledge, PDE1A and PDE1C transcripts are abundant in VSMCs [12], but only PDE1B is identified in macrophages. Indeed, knockdown of PDE1B in macrophages was associated with an increase in intracellular cGMP contents but without a concomitant decrease in Akt, IKKα/β and IκBα phosphorylation. In agreement with the findings of Jeon et al. [26], our results help to corroborate that the anti-inflammatory effects of vinpocetine are independent of a PDE1-mediated mechanism. These data together add our understanding that vinpocetine possess both PDE1-dependent and -independent properties, both of which play important role in the inhibition of atherosclerotic development [40].

In conclusion, the anti-atherogenic properties of vinpocetine observed in apoE^-/-^ mice may be explained by its inhibitory effects on monocyte adhesion, oxidative stress and inflammatory response mediated by Akt/NF-κB signaling pathway but independent of PDE1 blockade in macrophages. Our results provide new insights into the possible role of vinpocetine as a promosing therapeutic approach for atherosclerosis. 

Our study exists limitations that deserve further consideration. First, plasma levels of vinpocetine were unavailable in our experiments. Alternatively, we have carefully reviewed literatures with regard to pharmacokinetics of vinpocetine in clinical management of cerebrovascular disorders. The dosage of vinpocetine ranged from 1mg/kg to 10 mg/kg. Within the measured intervals, plasma concentration fell from about 70 to 10 μM rapidly [41]. Taken together, these data helped corroborate that the dosages of vinpocetine intraperitoneally injected into mice and used in the cell culture seem to be appropriate. Second, although vinpocetine has shown its multifunction *in vitro* and *in vivo* by previous studies and our studies, the drawbacks of apoE^-/-^ mice should be considered since it is not an accurate model of human atherosclerosis in light of plaque instability. That is to say, it requires vigorous clinical trials to elucidate its effects on atherosclerotic plaque in human beings.

## Supporting Information

Figure S1 **Effects of vinpocetine on HUVECs apoptosis.** Representative flow cytometry images (A) and statistical results (B) showed early and late apoptosis ratio of HUVECs pretreated with different concentrations of vinpocetine. Each experiment was repeated four times.
(TIF)

Figure S2 **Effects of vinpocetine on foam cell formation.** A. Foam cell formation was visualized by oil red o staining. B. Results were shown as fold changes in the proportions of oil red o-stained positive area compared with control (a. Control, b. ox-LDL, c. ox-LDL+vinp 15 μM, e. ox-LDL+vinp 30 μM, f. ox-LDL+vinp 50 μM).
(TIF)

Figure S3 **Effects of vinpocetine on ox-LDL-induced phosphorylation of p38, ERK1/2 and JNK1/2.**
(TIF)

Table S1 **Summary of pimer sequences used for RT-PCR.**
(DOC)
