# Supplementary figures and images for: Inhibitory Effects of Vinpocetine on the Progression of Atherosclerosis Are Mediated by Akt/NF-κB Dependent Mechanisms in apoE-/- Mice
Source: PLoS One. 2013 Dec 9;8(12):e82509. doi: 10.1371/journal.pone.0082509 (PMC3857260; doi:10.1371/journal.pone.0082509)

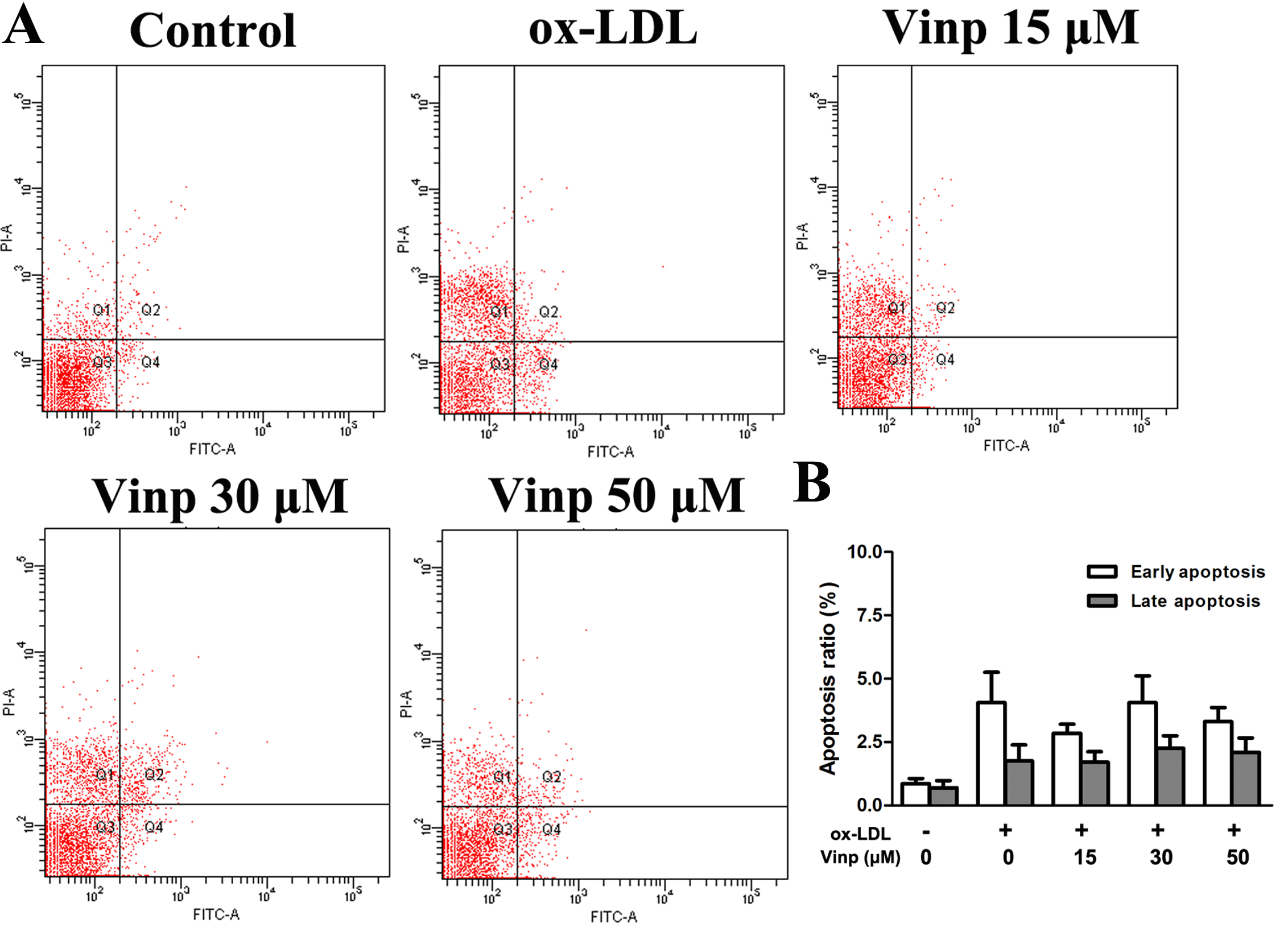

Supplement: Figure S1 — Effects of vinpocetine on HUVECs apoptosis. Representative flow cytometry images (A) and statistical results (B) showed early and late apoptosis ratio of HUVECs pretreated with different concentrations of vinpocetine. Each experiment was repeated four times. (TIF) [file pone.0082509.s001.tif]

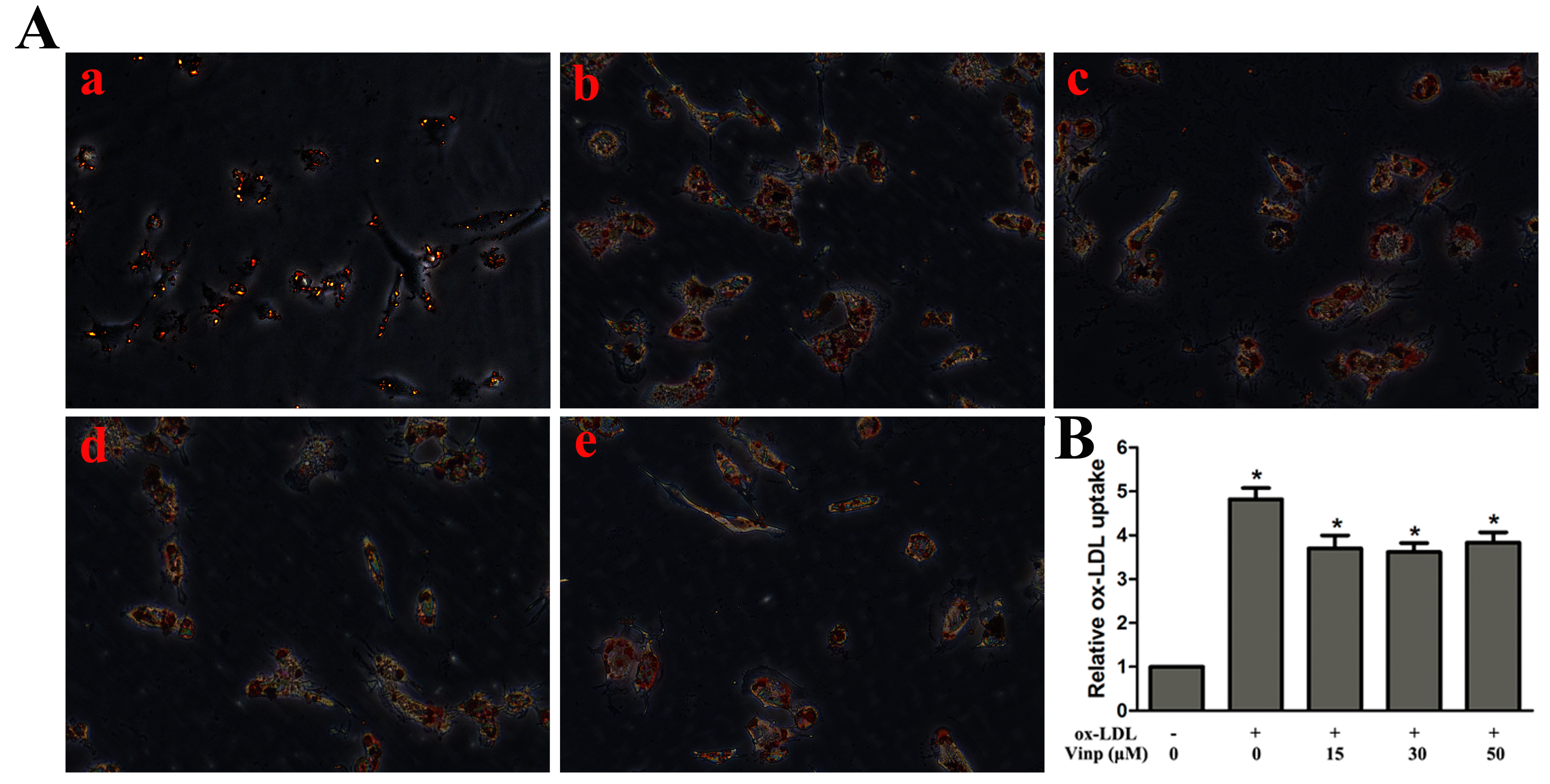

Supplement: Figure S2 — Effects of vinpocetine on foam cell formation. A. Foam cell formation was visualized by oil red o staining. B. Results were shown as fold changes in the proportions of oil red o-stained positive area compared with control (a. Control, b. ox-LDL, c. ox-LDL+vinp 15 μM, e. ox-LDL+vinp 30 μM, f. ox-LDL+vinp 50 μM). (TIF) [file pone.0082509.s002.tif]

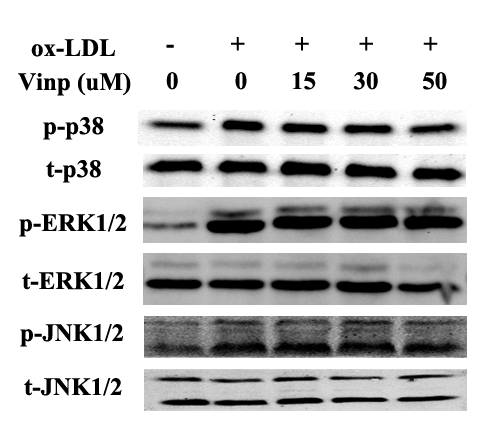

Supplement: Figure S3 — Effects of vinpocetine on ox-LDL-induced phosphorylation of p38, ERK1/2 and JNK1/2. (TIF) [file pone.0082509.s003.tif]
