# Supplementary material for: Inhibitory Effects of Vinpocetine on the Progression of Atherosclerosis Are Mediated by Akt/NF-κB Dependent Mechanisms in apoE-/- Mice
Source: PLoS One. 2013 Dec 9;8(12):e82509. doi: 10.1371/journal.pone.0082509 (PMC3857260; doi:10.1371/journal.pone.0082509)
Supplement: Table S1 — Summary of pimer sequences used for RT-PCR. (DOC) [file pone.0082509.s004.doc]

**Supporting Information**

**Table S1. Summary of pimer sequences used for RT-PCR**

| Gene | Primer sequence (5’→3’) |
| --- | --- |
| PDE1A | Forward: AGG TCA CTT CCA GCA AAT TA  Reverse: CCA CAT AGG AAG AAG TTT CG |
| PDE1B | Forward: GCT TTG ATG TCT TTT CCT TG  Reverse: ATT CTG ACT TGG TCT GGA TG |
| PDE1C | Forward: AGA TAT TAG CCA TCC AGC AA  Reverse: CAA CGG AGA TGA CAG AAT |
